# Alcohol consumption and risk of renal cell cancer: the NIH-AARP diet and health study

**DOI:** 10.1038/sj.bjc.6606089

**Published:** 2011-01-18

**Authors:** J Q Lew, W-H Chow, A R Hollenbeck, A Schatzkin, Y Park

**Affiliations:** 1 Division of Cancer Epidemiology and Genetics, National Cancer Institute, 6120 Executive Blvd., Bethesda, MD, USA; 2 St. Louis Children's Hospital, Washington University Medical Center, St. Louis, MO, USA; 3 AARP, Washington, DC, USA

**Keywords:** alcohol, renal cell cancer, cohort

## Abstract

**Background:**

The effect of moderate to heavy drinking (>15 g per day) on renal cell cancer (RCC) risk is unclear.

**Method:**

The relationship between alcohol consumption and RCC was examined in the NIH-AARP Diet and Health Study (*n*=49 2187, 1814 cases).

**Results:**

Compared with >0 to <5 g per day of alcohol consumption, the multivariate relative risk (95% confidence intervals) for 15 to <30 and ⩾30 g per day was, 0.75 (0.63–0.90) and 0.71 (0.59–0.85), respectively, in men and 0.67 (0.42–1.07) and 0.43 (0.22–0.84), respectively, in women.

**Conclusion:**

Alcohol consumption was inversely associated with RCC in a dose–response manner. The inverse association may be extended to ⩾30 g per day of alcohol intake.

As only a small proportion of renal cell cancer (RCC) arose from the milieu of familial cancer syndromes with culpable genetic mutations, environmental exposures and their interaction with genetic susceptibility are believed to have an important role in the development of sporadic RCC. However, except for cigarette smoking, hypertension, and obesity, few modifiable risk factors of RCC have been established (Chow *et al*, 2010).

Although alcohol has been positively associated with a variety of cancers, notably female breast cancer, cancers of the oral cavity, pharynx, oesophagus and larynx, colon, and liver (World Cancer Research Fund, 2007), a lower risk with alcohol consumption has been reported for cancers of thyroid and kidney (Allen *et al*, 2009). A pooled analysis of 12 prospective studies of RCC (1430 cases) (Lee *et al*, 2007) found a 29% and a 27% lowered risk in men and women, respectively, comparing drinkers of ⩾15 g per day of alcohol with non-drinkers. However, there was no further risk reduction with increasing level of drinking. Other recent studies also suggested inverse associations between alcohol consumption and RCC risk (Parker *et al*, 2002; Nicodemus *et al*, 2004; Rashidkhani *et al*, 2005; Setiawan *et al*, 2007; Hu *et al*, 2008; Pelucchi *et al*, 2008). However, due to limited ranges of alcohol consumption in most studies, including those in the pooled analysis, the effect of heavy drinking on RCC has not been examined fully.

To further clarify the association between alcohol drinking and risk of RCC, we examined data from a large prospective cohort that has 1814 RCC cases and a wide range of alcohol intake.

## Materials and methods

### Study population

Details of the NIH-AARP Diet and Health study design have been described elsewhere (Schatzkin *et al*, 2001). Briefly, the study was started in 1995–1996 when a food frequency questionnaire (FFQ) was mailed to members of AARP, aged 50–71 years and residing in one of six states (California, Florida, Pennsylvania, New Jersey, North Carolina or Louisiana) or two metropolitan areas (Atlanta (Georgia) or Detroit (Michigan)). Information regarding demographic and anthropometric characteristics and health-related behaviours were also solicited in the same mailing. The study was approved by the Special Studies Institutional Review Board of the US National Cancer Institute.

Among men and women who returned the questionnaires with satisfactory dietary data (*n*=566 402), we excluded individuals who were not the intended respondent, those who had any prevalent cancer other than non-melanoma skin cancer or had self-reported end-stage renal disease at baseline, participants who had cancer as a cause of death but no incidence report from cancer registries, participants who had died or moved out of the study area before their questionnaires were received, and participants who reported extreme energy intake. The analytic cohort consisted of 293 466 men and 198 721 women.

### Assessment of alcohol intake and other risk factors

We assessed diet including alcohol consumption using the FFQ, an earlier version of the National Cancer Institute's Diet History Questionnaire. To assess alcohol intake during the past year, the FFQ queried consumption of beer during the summer, beer during the rest of the year, liquor or mixed drinks, or wine or wine coolers during the entire year with 10 categories of frequency, ranging from ‘never’ to ‘⩾6 times per day’, and three portion sizes (<1, 1–2, >2 drinks). One drink of alcoholic beverage was defined as one 12-fluid-ounce beer, one 5-fluid-ounce glass of wine, or one 1.5-ounce shot of liquor equalling ∼13 g of alcohol. A study conducted in population similar to our study showed that alcohol intake assessed by a FFQ was highly correlated with intake measured by multiple week diet record (0.92 for men and 0.90 for women) (Giovannucci *et al*, 1991). We also collected demographic, anthropometric, and other cancer risk factors such as smoking, physical activity, and medical history at baseline.

### Identification of RCC cases

We identified RCC cases through linkage to the eight original state registries and two additional states subsequently added to capture participants who moved to those states (Arizona and Texas). We defined RCC as a first primary malignancy with the *International Classification of Diseases for Oncology*, 3rd edition, topography codes C649 and histology code 8140-8575.

### Statistical analysis

RCC risk in relation to alcohol drinking was assessed by estimating the relative risk (RR) and 95% confidence intervals (CIs) using the Cox proportional hazards regression model. Person-years of follow-up were calculated from the return date of the baseline questionnaire to the date of cancer diagnosis, death, emigration out of the study area, or end of follow-up (31 December 2006) whichever occurred first. We estimated RRs according to categories of alcohol consumption 0, >0 to <5 (reference), 5 to <15, 15 to <30, and ⩾30 g per day. The cut-points were decided based on *a priori* cut-points used in other studies. To test for linear trend across those categories, we assigned ordinal values for the categories and entered them as a continuous variable in the regression model. In the analysis of beer, wine, and liquor consumption, we mutually adjusted for the other types of alcoholic beverages in the model.

In multivariate models, we adjusted for age, race/ethnicity, education, marital status, body mass index (BMI), smoking history, vigorous physical activity, history of physician-diagnosed hypertension, and intakes of protein and total energy excluding calories from alcohol. We further adjusted for a history of diabetes, and hormone replacement therapy and oral contraceptive use in women and found that the results did not change materially. We created missing indicator category for missing values in each variable. The proportion of missing for each variable was generally <4%.

To identify potential effect modifiers, we tested alcohol–covariate interaction terms in relation to RCC using the likelihood ratio test with alcohol intake considered as a continuous variable. The covariates examined were sex, smoking status, history of physician-diagnosed hypertension, and BMI. We found a statistically significant interaction with sex (*P*=0.002), and therefore reported sex-specific results. SAS statistical software (version 9.1; SAS Institute, Inc., Cary, NC, USA) was used for all analyses. All statistical tests were two-sided, *P* values <0.05 were considered statistically significant.

## Results

During an average of 9 years and 4 476 544 person-years of follow-up, we identified 1814 RCC cases (1348 in men and 466 in women). During the year before the baseline questionnaire, 21% of men and 30% of women did not consume alcohol. The proportion of participants who consumed beer, wine, and liquor was 67, 61, and 57%, respectively, in men and 35, 60, and 47%, respectively, in women. Among those in the highest category of alcohol consumption (⩾30 g per day), men on average consumed 2.7 drinks per day of beer, 0.7 drink per day of wine, and 2.7 drinks per day of liquor; and women consumed 0.9 drink per day of beer, 1.2 drinks per day of wine and 2.3 drinks per day of liquor. Compared with drinkers of >0 to <5 g per day of alcohol, heavier drinkers tended to be white, non-Hispanic, current smokers, and had a higher education level (Table 1).

Alcohol intake was inversely associated with the risk of RCC in men (Table 2). Compared with men who drank >0 to <5 g per day, risk decreased from 0.82 for drinkers of 5 to <15 g per day to 0.75 and 0.71 for drinkers of 15 to <30 and ⩾30 g per day, respectively (*P* for trend 0.004). We further examined the association in the group with the highest intake of alcohol. Comparing 30 to <50 and ⩾50 g per day of alcohol consumption with >0 to <5 g per day, the multivariate RR was 0.83 (95% CI: 0.65–1.06, 71 cases) and 0.63 (95% CI: 0.50–0.79, 88 cases), respectively. Among alcoholic beverages examined, consumption of beer, but not wine or liquor, showed an inverse association with risk of RCC. When the associations were examined after excluding RCC cases diagnosed within the first 2 years of follow-up, the results did not change appreciably. Among women, alcohol intake was also associated with significantly lowered risk of RCC (Table 3). Compared with >0 to <5 g per day drinkers, risk decreased from 0.73 (95% CI: 0.52–1.03) for drinkers of 5 to <15 g per day to 0.43 (95% CI: 0.22–0.84) for drinkers of ⩾30 g per day. Wine and liquor consumption, but not beer, suggested an inverse association with RCC risk. The multivariate RR for an increment of 1 drink per day of alcohol drinks among drinkers was 0.96 (95% CI: 0.94–0.99) in men and 0.73 (95% CI: 0.60–0.88) in women.

When we examined the association in men and women combined, the multivariate RRs (95% CI) for non-drinkers, 5 to <15, 15 to <30, and ⩾30 g per day of alcohol *vs* >0 to <5 g per day of alcohol were 0.91 (0.81–1.02), 0.80 (0.69–0.92), 0.74 (0.62–0.87), and 0.69 (0.58–0.82), respectively. The association between alcohol consumption and RCC did not differ by follow-up periods. In restricting analysis to cases identified during the first 2 years of follow-up (288 cases), the multivariate RRs (95% CI) for 15 to <30 and ⩾30 g per day of alcohol *vs* >0 to <5 g per day were 0.74 (0.49–1.12) and 0.58 (0.37–0.90), respectively. The corresponding RRs (95% CI) in analysis excluding cases occurred the first 2 years of follow-up (1526 cases) were 0.74 (0.62–0.89) and 0.71 (0.59–0.86), respectively.

We also examined whether the association between alcohol consumption and risk of RCC was modified by BMI, smoking status, and history of hypertension and found that the associations were not significantly modified by these risk factors (data not shown).

## Discussion

In this large prospective cohort study of adults aged 50 years and over, we found that the alcohol consumption was inversely associated with the risk of RCC in men and women. Our finding is consistent with previous studies that mostly examined the effect of light to moderate alcohol consumption. Investigations published after a pooled analysis of 12 prospective studies (Lee *et al*, 2007) also observed an inverse association between alcohol consumption and RCC risk. The Multiethnic Cohort Study (Setiawan *et al*, 2007) reported more apparent inverse association in men than women. Compared with non-drinkers, consumption of >1 drink per day was associated with a significant 31% risk reduction in men and a non-significant 20% risk reduction in women. Recently, the Million Women Study from the UK (Allen *et al*, 2009) also found a 34% lowered risk of RCC among women who drank ⩾15 drinks per week compared with women drinking ⩽2 drinks per week. Our study was able to examine the association with moderate to heavy alcohol consumption (⩾30 g per day) and found that RCC risk decreased linearly as alcohol consumption increased. Our study observed more apparent association of alcohol with RCC in women than in men. This may be partially due to differences in alcohol metabolism between men and women; studies suggested that women reach higher blood alcohol concentrations than men for same amount of alcohol per body weight and have greater clearance of alcohol per unit of lean body mass than men (Mumenthaler *et al*, 1999). However, we cannot rule out the possibility that the stronger association in women was a chance finding due to relatively small number of cases in women.

The relationship between different types of alcoholic beverages and RCC has been inconsistent (Parker *et al*, 2002; Nicodemus *et al*, 2004; Mahabir *et al*, 2005; Rashidkhani *et al*, 2005; Lee *et al*, 2006, 2007; Greving *et al*, 2007; Setiawan *et al*, 2007; Hu *et al*, 2008; Pelucchi *et al*, 2008). The inconsistent results with alcoholic beverages may be due to lack of power to examine individual alcoholic beverages, given small number of cases and narrow ranges of consumption for each alcohol beverage. Nevertheless, this observation may suggest that alcohol *per se*, rather than other constituents in individual alcoholic beverage, is likely responsible for the inverse association with RCC. More apparent association observed with beer in men and with wine in women in our study is partially because these are the drinks most commonly consumed in men and women.

Several mechanisms by which alcohol may reduce the risk of RCC have been proposed. It includes (a) improved insulin sensitivity (Facchini *et al*, 1994; Lazarus *et al*, 1997); (b) reduction in oxidative stress as alcoholic beverages contain anti-oxidant phenolic compounds and can remove oxidised carcinogenic agents, reduce lipid peroxidation and cell proliferation, and promote apoptosis (Gago-Dominguez *et al*, 2002; Lee *et al*, 2006; Lowrance *et al*, 2010); and (c) the diuretic effect of alcohol, which may help to control hypertension (Lee *et al*, 2006). However, increase in total fluid intake has not been established to influence RCC risk (Lee *et al*, 2006; Hu *et al*, 2009).

The strengths of our study include a prospective study design that minimises recall bias, a large number of RCC cases and a wide range of alcohol consumption, which allowed us to examine the effect of alcohol not only in moderate drinkers but also in heavy drinkers. Our study also has several limitations. Because alcohol consumption was assessed only at baseline in our study, we were not able to examine the effect of lifetime alcohol consumption or changes in drinking patterns on RCC risk. Also, because the never drinkers were defined as people who did not drink in the past year, it is possible that former heavy drinkers who might have stopped recently were classified as non-drinkers. To avoid this misclassification, we used >0 to <5 g per day of alcohol as a reference group.

In conclusion, we found a significant inverse association between alcohol consumption and risk of RCC in men and women. The association appears linear, with no threshold effect among heavy drinkers.

## Figures and Tables

**Table 1:** Selected characteristics of the study participants by alcohol consumption

	**Alcohol (g per day)**				
	**0**	**>0 to <5**	**5 to <15**	**15 to <30**	**30+**
*Men (*n)	61 289	100 074	51 022	39 676	41 405
Beer consumption (drinks per day)	0	0.1	0.3	0.5	2.7
Wine consumption (drinks per day)	0	0.1	0.2	0.5	0.7
Liquor consumption (drinks per day)	0	0.03	0.2	0.6	2.7
Age (years)	62	62	62	63	62
White, non-Hispanic (%)	90	92	93	95	95
Body mass index (kg m^−2^)	27.5	27.6	27.0	26.7	27.0
Married (%)	84	86	86	85	82
College graduate (%)	35	43	50	53	47
Physical activity (5+ times per week, %)	22	20	21	24	22
Current smoker (%)	10	9	9	10	17
Hypertension (%)	42	39	37	39	46
Protein (% of energy intake)	16	16	16	17	17
Energy intake^a^ (kcal per day)	1942	1864	1859	1866	1985
					
*Women (*n)	58 874	93 089	24 889	12 787	9082
Beer consumption (drinks per day)	0	0.02	0.1	0.2	0.9
Wine consumption (drinks per day)	0	0.05	0.4	0.5	1.2
Liquor consumption (drinks per day)	0	0.03	0.2	0.6	2.3
Age (years)	62	62	62	62	62
White, non-Hispanic (%)	85	90	93	95	94
Body mass index (kg m^−2^)	27.9	27.0	25.2	25.0	25.2
Married (%)	43	44	47	47	45
College graduate (%)	23	30	38	37	35
Physical activity (5+ times per week, %)	16	15	19	19	17
Current smoker (%)	12	13	15	20	29
Hypertension (%)	43	35	31	33	39
Protein (% of energy intake)	16	16	16	17	17
Energy intake^a^ (kcal per day)	1576	1521	1489	1484	1502

a Energy intake excluding calories from alcohol.

**Table 2:** Relative risks and 95% confidence intervals of renal cell cancer for alcohol and alcoholic beverage consumptions in men

	**Alcohol (g per day)**					
	**0**	**>0 to <5**	**5 to <15**	**15 to <30**	**30+**	***P* trend**
*Total alcohol*						
Cases (*n*)	275	543	216	155	159	
Age adjusted	0.84 (0.73–0.97)	1.00	0.78 (0.66–0.91)	0.70 (0.59–0.84)	0.72 (0.61–0.86)	<0.001
Multivariate^a^	0.83 (0.71–0.96)	1.00	0.82 (0.70–0.96)	0.75 (0.63–0.90)	0.71 (0.59–0.85)	0.004
						
	**0**	**>0 to <5**	**5 to <15**	**15+**	***P* trend**	
*Beer*						
Cases	463	695	113	77		
Age adjusted	0.99 (0.88–1.12)	1.00	0.79 (0.65–0.96)	0.64 (0.51–0.81)	<0.001	
Multivariate	0.97 (0.86–1.09)	1.00	0.81 (0.66–0.99)	0.63 (0.49–0.80)	<0.001	
						
*Wine*						
Cases	550	607	101	90		
Age adjusted	1.06 (0.95–1.19)	1.00	0.87 (0.70–1.07)	0.88 (0.70–1.10)	0.02	
Multivariate	0.99 (0.88–1.11)	1.00	0.95 (0.77–1.17)	0.97 (0.77–1.21)	0.82	
						
*Liquor*						
Cases	570	565	57	156		
Age adjusted	0.94 (0.84–1.06)	1.00	0.84 (0.64–1.10)	0.90 (0.75–1.08)	0.62	
Multivariate	0.92 (0.82–1.04)	1.00	0.84 (0.64–1.10)	0.87 (0.73–1.04)	0.59	

a Multivariate model adjusted for age, race (white, non-Hispanic, black, non-Hispanic, and others), body mass index (<25, 25–<30, and ⩾30), marital status (married: yes and no), education (less than high school, high school graduate, some college, and college/post college), vigorous physical activity (never/rarely, 1–3 times per month, 1–2, 3–4, and ⩾5 times per week), smoking (never, past ⩽20 cigarettes per day, past >20 cigarettes per day, current ⩽20 cigarettes per day, and current >20 cigarettes per day), history of hypertension (yes and no), and intakes of protein (quintiles) and total energy excluding energy from alcohol (continuous).

**Table 3:** Relative risks and 95% confidence intervals of renal cell cancer for alcohol and alcoholic beverage consumptions in women

	**Alcohol (g per day)**					
	**0**	**>0 to <5**	**5 to <15**	**15 to <30**	**30+**	***P* trend**
*Total alcohol*						
Cases (*n*)	171	227	40	19	9	
Age adjusted	1.18 (0.97–1.44)	1.00	0.66 (0.47–0.92)	0.61 (0.38–0.97)	0.42 (0.22–0.82)	<0.001
Multivariate^a^	1.07 (0.87–1.31)	1.00	0.73 (0.52–1.03)	0.67 (0.42–1.07)	0.43 (0.22–0.84)	<0.001
						
	**0**	**>0 to <5**	**5+**	***P* trend**		
*Beer*						
Cases	333	123	10			
Age adjusted	1.33 (1.08–1.64)	1.00	0.95 (0.50–1.82)	0.006		
Multivariate	1.14 (0.92–1.42)	1.00	0.95 (0.50–1.82)	0.21		
						
*Wine*						
Cases	226	204	36			
Age adjusted	1.32 (1.09–1.59)	1.00	0.67 (0.47–0.95)	<0.001		
Multivariate	1.12 (0.92–1.37)	1.00	0.78 (0.55-1.12)	0.05		
						
*Liquor*						
Cases	283	156	27			
Age adjusted	1.34 (1.10–1.63)	1.00	0.83 (0.55–1.24)	<0.001		
Multivariate	1.21 (0.99–1.48)	1.00	0.85 (0.56–1.29)	0.03		

a Multivariate model adjusted for age, race (White, non-Hispanic, Black, non-Hispanic, and others), body mass index (<25, 25 to <30, and ⩾30), marital status (married: yes and no), education (less than high school, high school graduate, some college, and college/post college), vigorous physical activity (never/rarely, 1–3 times per month, 1–2, 3–4, and ⩾5 times per week), smoking (never, past ⩽20 cigarettes per day, past >20 cigarettes per day, current ⩽20 cigarettes per day, and current >20 cigarettes per day), history of hypertension (yes and no), and intakes of protein (quintiles) and total energy excluding energy from alcohol (continuous).
